# Comparative Study of Different Combinational Mucoadhesive Formulations of Sumatriptan-Metoclopramide

**DOI:** 10.15171/apb.2016.018

**Published:** 2016-03-17

**Authors:** Mitra Jelvehgari, Hadi Valizadeh, Sanam Ziapour, Mahdieh Rahmani, Seyed Hassan Montazam, Saieede Soltani

**Affiliations:** ^1^ Drug Applied Research Center, Tabriz University of Medical Sciences, Tabriz, Iran.; ^2^ Faculty of Pharmacy, Tabriz University of Medical Sciences, Tabriz, Iran.; ^3^ Biotechnology Research center, Tabriz University of Medical Sciences, Tabriz, Iran.; ^4^ Student Research Committee, Tabriz University of Medical Sciences, Tabriz, Iran.; ^5^ Department of Microbiology, Bonab Branch, Islamic Azad University, Bonab, Iran.

**Keywords:** Mucoadhesive, Buccal, Transmucosal, Sumatriptan, Metoclopramide

## Abstract

***Purpose:*** Sumatriptan succinate (Sum) is a Serotonin 5- HT1 receptor agonist, used in the treatment of migraine. It is absorbed rapidly but incompletely when taken orally and underwent first - pass metabolism, resulting in a low bioavailability of about 15%. The aim was to design mucoadhesive buccal discs and sublingual films of Sum and metoclopramide (Met) combined to improve their bioavailability.

***Methods:*** In the current study, the microparticles and films were prepared by emulsion solvent diffusion (ESD) and solvent casting methods, respectively. Buccal-mucoadhesive microparticles and films with different drug to polymer ratios were prepared and characterized by encapsulation efficiency, particle size, DSC (Differential Scanning Calorimetric), folding endurance, mucoadhesive property and drug release studies.

***Results:*** The best drug/s to polymer ratios in films and microparticles were 1:2.7:8 (SM_2_) and 1:4:6 (SM_4_), respectively. The film of SM_2_ showed 11.01 mg weight, 123 µm thickness and 300 folding endurance. The production yield was 107.33% for SM_4_ microparticles, 323.59 µm for mean particle size and 94.53% for loading efficiency (for Sum) and 104.18% (for Met). The DSC showed no stable characteristic of Sum and Met in the drug loaded films/discs and revealed amorphous form and transition of hydrate to anhydrous form for Met. The films exhibited very good mucoadhesive properties and shorter retention time (15-30 s) in comparison with the discs (130 min). The results showed that the discs prepared had slower release than the films (*p*<0.05).

***Conclusion:*** Films and discs of Sum-Met combinations were successfully prepared with improved release and mucoadhesive properties.

## Introduction

Migraine is recognized as a common neurovascular disorder which displays recurrent episodes of immobilizing headache and autonomic nervous system dysfunction. Neurological aura symptoms are also observed in some patients.^1,2^ Nonspecific treatments such as antiemetics are prescribed in treating any kind of pain, and specific therapies such as triptans are exclusively effective in treating migraine and related disorders.^3,4^ Triptans are frequently prescribed in the treatment of acute migraine attacks as they are potent in providing wide efficacy and tolerability.^1,5^

Sumatriptan succinate (Sum) as a 5-HT1receptor agonist is employed in the removal of symptoms of migraine headaches. However, due to severe nausea or vomiting during the migraine attacks and low oral bioavailability (15%) because of high first-pass metabolism, the oral treatment proves unsatisfactory.^6,7^

This medication is commercially available as oral tablet, nasal spray and subcutaneous injection.^8^

The antiemetic properties of metoclopramide (Met) apparently are a result of antagonism of its central and peripheral dopamine receptors. Met is ordinarily prescribed for the treatment of nausea and vomiting. This drug is highly water soluble and is swiftly absorbed after oral administration. It has a short biological half-life (4.5 h) and is commonly administered in a dose of 10 to 15 mg four times per day in order to maintain effective concentrations throughout the day. In long -term therapy, fluctuation in the plasma concentration, with high concentration peaks are common for drugs with rapid absorption and elimination.

Hydroxypropyl methylcellulose is an odorless and tasteless, white to slightly off-white, fibrous or granular, free-flowing powder that is a synthetic modification of the natural polymer, cellulose. The reason for its widespread acceptance include (1) solubility characteristics of the polymer in gastrointestinal fluid, and in organic and aqueous solvent systems, (2) noninterference with tablet disintegration and drug availability, (3) flexibility, chip resistance and absence of taste and odor, (4) stability in the presence of heat, light, air or reasonable levels of moisture, (5) ability to incorporate color and other additives into the film without difficulty.

Thin-film and disc-buccal drug delivery benefit a dissolving film or oral drug to be administered via absorption in the mouth (buccally or sublingually) and/or in small intestines (enterically). Film is prepared using hydrophilic polymers that rapidly dissolve upon the tongue or buccal cavity, and delivers the drug to the systemic circulation through dissolution when a contact is made with liquid. Thin dissolvable films of drugs delivered sublingually are potent to trigger the beginning of action, lower the dosing, and increase the efficacy and safety profile of the medication.^9^

Therefore the objectives of this work are; to develop a new drug delivery system for Sum which could be administered through the buccal (discs prepared from microspheres)/sublingual (film) route, to particularly target the mucous region in order to result in a rapid relief by fast onset of action; to increase bioavailability; and to avoid the second dose administration. Its sublingual film and buccal disc applications may be better alternatives that also reduce the side effects associated with oral and parental therapy.^7^

Oral transmucosal absorption generally occurs rapidly because of the rich vascular supply to the mucosa and the lack of a stratum corneum epidermidis. This minuscule barrier to drug transport results in a rapid rise in blood concentration level. Oral transmucosal administration has the advantage in avoiding the enterohepatic circulation and instaneous destruction by gastric acid or partial first-pass effects of hepatic metabolism.^10^ Sublingual and buccal medications are prepared either in the form of small, quick dissolving tablets, or in sprays, lozenges and liquid suspensions. The administration of these medications is through placing the medication in the mouth, either under the tongue (sublingual), or between the gum and the cheek (buccal). This form of medication administration is very adequate as it bypasses the digestive system and is absorbed in the blood flow in minutes. Should administered properly, sublingual and buccal therapies act within 1 to 5 min of administration.^11^

Hence the present study aimed to prepare and evaluate sublingual film/buccal discs of sumatriptan combined with methoclopramide and using bioadhesive polymer, in order to improve the therapeutic efficacy of these drugs and reduce their dose dependent side effects and frequency of administration in treating migraine.

## Materials and Methods

### Materials 

Sumatriptan succinate (Sum), metoclopramide hydrochloride (Met), HPMC (E-15), ethanol, dichloromethane, potassium dihydrogen phosphate, sodium hydroxide, sodium chloride, potassium chloride, sodium sulfate, ammonium acetate, urea, lactic acid, liquid paraffin, span 80 and propylene glycol were obtained from Merck (Darmstadt, Germany). All solvents and reagents were of analytical grade.

### Experimental methods

#### Preparation of Sum and Met combined film

Sublingual films of Sum and Met combined were prepared by solvent casting technique using a film forming mucoadhesive polymer. HPMC was accurately weighed (200 mg) and dissolved in 2.5 ml of ethanol. The beaker containing polymer and ethanol was kept aside for 5 min for the polymer to swell. Next, 2.5 ml of dichloromethane was added to the above polymer solution and the dispersion was stirred. Then, one drop of propylene glycol (0.030 g) was added to the polymer solution. Sum drug was accurately weighed with a different drug-to-polymer ratio (50, 66.7 and 100 mg) and then dissolved in 2 ml of water in beaker. In second step, Met drug was weighed with a constant amount (25 mg) and added to the Sum solution beaker. The drugs' solution was then added to the polymer solution and mixed thoroughly with a magnetic stirrer. The solution was utterly poured into the glass Petri dish placed over a flat surface. Then an inverted funnel was placed over the dish to prevent sudden evaporation. The mould containing the polymeric solution of drug was kept for 12 h at room temperature to dry. After drying, the films were observed and checked for possible imperfections upon their removal from the moulds. They were covered with wax paper and kept in desiccators until the evaluation tests were conducted. These new films were examined in order to identify and select the film showing the best characteristics (Table 1).

**Table 1:** Sumatriptan succinate combined with methoclopramide hydrochloride films/discs with different drug to polymer ratios prepared by solvent casting and (O1/O2) emulsion solvent diffusion method, respectively

**Formulation code**	**Drug to polymer ratio**	**Metoclopramide hydrochloride** **(mg)**	**Sumatriptan succinate** **(mg)**	**HPMC** **(mg)**	**water** **(ml)**	**dichloromethane** **(ml)**	**ethanol** **(ml)**	**Liquid paraffine** **(ml)**	**Petrulum ether** **(ml)**	**Span 80** **(g)**	**Propylen glycol** **(g)**
SM_1_	1:2:8	25	50	200	2	2.5	2.5	-	-	-	0.03
SM_2_	1:2.7:8	25	66.7	200	2	2.5	2.5	-	-	-	0.03
SM_3_	1:4:8	25	100	200	2	2.5	2.5	-	-	-	0.03
SM_4_	1:4:6	50	200	300	-	2.5	2.5	100	25	3	0.03
SM_5_	1:4:10	50	200	500	-	2.5	2.5	100	25	3	0.03
SM_6_	1:4:14	50	200	700	-	2.5	2.5	100	25	3	0.03

#### Preparation of Sum and Met combination microparticles

Microparticles of Sum and Met were prepared using HPMC polymer with different drugs-to-polymer ratios (1:4:6, 1:4:10 and 1:4:14 w/w). Briefly, HPMC (300, 500 and 700 mg) was dissolved in 2.5 ml of ethanol and 2.5 ml of dichloromethane by stirring at 500 rpm with magnetic stirrer. Drugs were then added to polymeric solution and stirred until complete dissolution. Then the resultant drug-polymer suspension was injected using a 5 ml syringe into 100 ml of light liquid paraffin with 3 g of span 80 and propylene glycol (0.030 g) while stirring to form an O1/O2 emulsion. Stirring was continued for 2 h and then 25 ml of petroleum ether was added at 600 rpm until complete solvent evaporation and microspheres’ formation. After another 2 h, the hardened microparticles were collected by filtration and washed with three portions of 30 ml petroleum ether and air dried at room temperature for 24 h. Microspheres were allowed to be dried at room temperature (Table 1).

### Characterization of buccoadhesive microparticles’ studies

#### Determination of loading efficiency and production yield 

The loading efficiency was calculated using the following formula:

Loading efficiency (%) = (actual drug content in microparticles/theoretical drug content) × 100 Eq. (A-1)

The production yield of the microparticles was determined by dividing the final weight of the polymeric particles to the initial weight of the raw materials. Each determination was performed in triplicate.

#### Frequency distribution analysis

Samples of microparticles were analyzed for frequency distribution with calibrated optical microscope, fitted with a stage and an ocular micrometer. Small quantities of microsphere were spread on a clean glass slide and the average particle size of 60, frequency distribution and mean particle size were determined in each batch using scion image and sigma plot software packages.

#### Disc production and physicochemical characterization

Each disc contained 100 mg of Sum and Met microspheres (with different drug-to-polymer ratios of 1:4:6, 1:4:10, 1:4:14). The discs were round and flat with an average diameter of 6 ± 0.1 mm compressed with a constant compression force (2 tones). Hardness of the discs was determined for six discs using Erweka hardness tester (Erweka, Germany). Friability of the prepared discs was assessed using friability tester (Erweka, Germany).

### Characterization of buccoadhesive film studies

Appearance of the films was appraised by observing the color, elegance, stickiness and texture.

#### Weight uniformity of films

Six films of size 1×1cm^2^ of every formulation were individually weighed in a digital balance (Sartorius, Germany) and the weight variation was calculated.

#### Thickness uniformity of the films

Thickness of films was measured using digital vernier calipers at five different points (one center and four corners) of the film and the average was calculated (Mitutoyo, Japan).^12^

#### Folding endurance

The folding endurance of each film was decided by counting the number of times the film (size 1x1 cm^2^) could be folded repeatedly (folded or broken up to 300 times), which was supposed reasonable to reveal good film properties.^13^

#### Moisture content loss and moisture absorption

The films were accurately weighed and kept in desiccators containing: a) anhydrous calcium chloride and b) 100ml of saturated solution of aluminum chloride, which maintained 76% and 86% humidity (RH). After 3 days, the films were taken out and weighed. The moisture content (%) was determined by calculating the moisture loss (%) using the following formula:^13^

Moisture content (%) =Initial weight – final weight / Initial weight × 100 Eq. (A-2)

The moisture absorption was also calculated using the following formula:^13^

Moisture absorption (%) = Final weight - initial weight / Initial weight ×100 Eq. (A-3)

#### Drug content and content uniformity

The films (five samples of each film) were analyzed for the content uniformity by dissolving 1×1cm^2^ films in 10 ml phosphate buffer, pH 6.8, with simultaneous shaking for several hours. The absorbance of the solution (Sum and Met) was measured by UV spectrophotometer at 227.4 (Sum) and 272.4 (Met) nm. All experiments were performed in triplicate.

### Characterization of buccoadhesive film/microparticles’ studies

#### Differential Scanning Colorimetry (DSC)

The physical state of drug in the microspheres was analyzed by Differential Scanning Calorimeter (Shimadzu, Japan). The thermograms were obtained at a scanning rate of 10 °C/min conducted over a temperature range of 25-300 °C.

#### Determination of surface pH 

The surface pH of formulations was determined using a combined glass electrode in order to investigate their possible side effects *in vivo.* An acidic or alkaline formulation causes the irritation of mucosal membrane and hence this is an important parameter in developing a mucoadhesive dosage form. The discs/films were first allowed to swell by keeping them in contact with 5 ml phosphate buffer and pH 6.8 for 4 h in 50 ml beakers. pH was then noted by bringing the electrode near the surface of the formulation and allowing equilibration for 1 min. Surface pH was measured at predetermined time intervals (0, 60, 90, 120, and 240 min). The experiments were carried out in triplicate.

#### Disc swelling studies

Bioadhesive preparations can swell in the presence of saliva. The swelling rate of buccoadhesive discs was evaluated by placing the accurately weighed discs (W_1_) in 50 ml phosphate buffer solution (pH 6.8) at 37 °C. Swelling was measured at predetermined time intervals (15, 30, 60, 90 and 120 min). Then the discs were removed from the beaker after carefully removing the excess surface water using the filter paper. The swollen disc was weighed again (W_2_) and the swelling index were calculated accordingly:

Swelling index = (W_2_- W_1_)/ W_1_ × 100 Eq. (B-1)

Initial diameter of the film (1x1 cm^2^) was determined when placed in a phosphate buffer solution and incubated at 37±1°C was (D_1_). Then swollen film diameter was re-measured (D_2_) at regular intervals (up to 1 h) and the swelling index was calculated using the following formula:^14,15^

Swelling index = D_2_-D_1_/D_1_ Eq. (B-2)

#### Ex vivo mucoadhesion time

Sheep were used in this study. The animals were fed and watered ad libitum. They were kept in the Animal House at a controlled ambient temperature of 25±2 °C with 50±10% relative humidity and a 12-h light/ 12-h dark cycle. The present study was conducted according to the Guide for the Care and Use of Laboratory Animals of Tabriz University of Medical Sciences, Tabriz-Iran (National Institutes of Health Publication No 85-23, revised 1985). The selected film/discs were subjected to *ex vivo* mucoadhesion test. The disintegration medium contained 900 ml phosphate buffer, pH 6.8, maintained at 37 °C. A segment of sheep buccal mucosa, 3 cm long, was glued to the surface of a glass slab and vertically attached to the disintegration apparatus (Erweka, Germany).^16^ The mucoadhesive films/discs were hydrated from one surface and then brought into contact with the mucosal membrane. The glass slab was so vertically fixed to the apparatus that allowed it to move up and down; thus, the film was completely immersed in the buffer solution at the lowest point and was out at the highest point. The time demanded for complete erosion or detachment of the films from the mucosal surface was recorded. The experiment was performed in triplicate.

#### Bioadhesion strength

**Figure 1:**
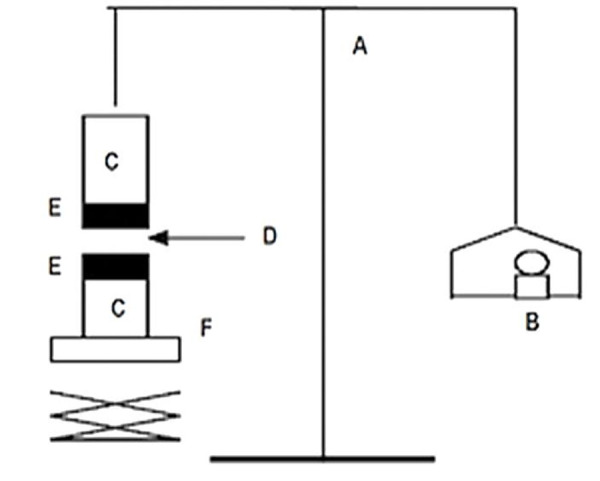
Bioadhesive force measuring device: (A) modified balance; (B, E) glass vial; (C) discs; (D) tissue; (F) Weights; (G) height-adjustable pan.

The tensile strength required to detach the bioadhesive films/discs from the mucosal surface was applied as a measure of the bioadhesive performance. The apparatus was locally assembled. The device mainly composed of a two-arm balance (Figure 1).

The mucoadhesive forces of films were measured by means of the mucoadhesive force-measuring device,^16^ using the tissue cut from sheep buccal mucosa. The pieces of mucosa were stored frozen in phosphate buffer, pH 7.4, and thawed to room temperature before use. At the time of testing, a section of mucosa was secured to the upper glass vial (C) using a cyanoacrylate adhesive (E). The diameter of each exposed mucosal membrane was 1.5 cm. The vials were equilibrated and maintained at 37 °C for 10 min. Next, one vial with a section of tissue (E) was connected to the balance (A) and the other vial was fixed on a height-adjustable pan (F). To expose the tissue on this vial, a constant amount of films (D) was applied. The height of vial was so adjusted that the films/discs could adhere to the mucosal tissues of both vials. Immediately, a constant force of 0.5 N was applied for 2 min to ensure the intimate contact between the tissues and the samples. The vial was then moved upwards at a constant speed and connected to the balance. Weights were added at a regular rate to the pan on the other side of the modified balance of the used device until the two vials were separated. During measurement, 150 μl of phosphate buffer at pH 6.8 was evenly spread onto the surface of the test membrane. The bioadhesive force, noticed as the detachment stress in g/cm^2^, was determined from the minimal weights that detached the tissues from the surface of each formulation using the following equation:^16^

Eq. (B-3) $$\text{Detachment Stress }\left({\text{g/cm}}^{\text{2}}\right)\text{ =}\frac{\text{m}}{\text{A}}$$

Where *m* is the weight added to the balance in grams and *A* is the area of tissue exposed. Measurements were repeated three times for each of the films. All the above three experiments were conducted in triplicate.

#### Permeation studies

The *in vitro* permeation study of the Sum and Met combined films/discs through the buccal mucosal of sheep was performed using Franz diffusion cell at 37 ± 0.2 °C. Freshly obtained buccal mucosa was mounted between the donor and receptor compartments so that the smooth surface of the mucosa faced the donor compartment. The films/discs were placed on the mucosa and the compartments were clamped together. The donor compartment was filled with 3 ml simulated saliva, pH 6.8 (sodium chloride 4.50 g, sodium sulfate 0.30 g, potassium chloride 0.30 g, urea 0.20 g, ammonium acetate 0.40 g, lactic acid 3 g, and distilled water up to 1,000 mL, adjusting pH of the solution to 6.8 by 1 M NaOH solution). The receptor compartment was filled with 22-25 ml phosphate buffer, pH 7.4, and stirred with a magnetic bead at 700 rpm.^17^

Three milliliters of sample were withdrawn at predetermined time intervals and analyzed for drugs at 228 (Sum) and 272.4 (Met) nm.

#### *In vitro* release studies

*In vitro* release studies were carried out using an incubator shaker at 37 ±0.5 °C, at a stirring speed of 50 rpm. Films/discs were fixed on glass slides and placed at the bottom of beaker. The studies were performed for all formulations (Sum and Met combination) in triplicate, using 50 ml (37 °C, 50 rpm) of isotonic phosphate buffer (pH 6.8) as the dissolution medium. An aliquot of 3 ml sample was withdrawn at regular intervals and replaced immediately with an equal volume of fresh phosphate buffer (pH 6.8). Samples were then analyzed at 226.6 (Sum) and 272 (Met) nm with UV spectrophotometer.

#### Histopathological evaluation of mucosa

Histopathological evaluation of tissue incubated in phosphate buffer, pH 6.8, was compared with that treated with sublingual mucoadhesive films/discs delivered from mucoadhesion time test. The tissue was fixed with 10% formalin, routinely processed, and embedded in paraffin. The sections were cut and stained with hematoxylin and eosin on glass slides. A pathologist, blinded to the study, worked on detecting any damage to the tissue and assessed the sections on the light microscope.^17^

## Results and Discussion

Mucoadhesive drug delivery system is a high potential means that is applied in delivering drugs to the body through targeting the stratified squamous epithelium which is supported by a connective tissue lamina propria in buccal mucosal membrane.^18^ Drug penetration into the membrane first leaves behind a net of capillaries and arteries in lamina propria and then reaches the systemic circulation through the internal jugular vein.^19^ This drug delivery system benefits from bioadhesion property of determined water soluble polymers which become adhesive on hydration and hence could be used in targeting buccal mucosa (lining of the cheek) to the systemic circulation.^20^ The mucoadhesive drug delivery system suggests apparently some advantages. For example, the system enhances drug bioavailability as a result of avoidance of first-pass metabolism; if it encounters toxicity, effortlessly terminates the therapy and removes the dosage form from the buccal cavity; this system requires less frequency of administration and therefore shows a better patient compliance; it significantly reduces the costs and dose-related local or systemic side effects by targeting the disease sites or tissues; and in steady-state levels, it shows a reduction in fluctuation.^21^ Thus, adhesive mucosal dosage forms have been prepared in the form of adhesive tablets (discs), adhesive gels, and adhesive patches (films) for oral delivery.^22^ In transmucosal delivery, the demanded quantity of mucoadhesive polymer was supplied with required volume of solvent system and vortexed to allow the polymer to swell. The required quantity of drug was also dissolved in a small volume of solvent system and added to the polymer solution and mixed well.

In the present study, we prepared the sublingual films and mucoadhesive microspheres containing Sum and Met combinations by solvent casting technique and evaporation/extraction technique, respectively. The drug delivery system was formulated as a matrix. The physicochemical and mucoadhesive characteristics of all the formulations are shown in Table 2.

**Table 2:** Effect of drug to polymer ratio on physicochemical characteristics and mucoadhesivity of sumatriptan combined with methoclopramide films/discs

**Variables**	**Formulation code**					
	**SM**_1_	**SM**_2_	**SM**_3_	**SM**_4_	**SM**_5_	**SM**_6_
**Met :Sum: HPMC ratio**	1:2:8	1:2.7:8	1:4:8	1:4:6	1:4:10	1:4:14
**Weight variation** **(mg ± SD)**	9.50 ± 2.50	11.01 ± 0.80	14.02 ± 1.20	99.85 ± 2.50	100.01 ± 2.80	99.02 ± 1.20
**thickness** **(µm± SD)**	110.30 ± 0.03	123 ± 0.007	243 ± 0.05	-	-	-
**Folding endurance** **(n±SD)**	>300±7	>300±10	89 ± 12	-	-	-
**Drug content** **(%±SD)**	**Sum:** 1.03 ± 0.33	1.15 ± 0.60	0.23 ± 0.02	15 ± 0.06	16.70 ± 0.23	19.90 ± 0.1
	**Met:** 0.59 ± 0.50	0.60 ± 0.80	0.57 ± 0.74	3.80 ± 0.06	4.30 ± 0.06	5.48 ± 0.06
**Loading Efficiency** **(%±SD)**	**Sum: ** -	-	-	94.53±3.58	62.64±1.58	41.25±1.32
	**Met: **-	-	-	104.18±2.33	64.56±3.75	41.80±1.06
**Content uniformity** **(%±SD)**	**Sum:** 100 ± 0.02	98.59 ± 0.02	96.63 ±0.02	100 ± 0.12	97.49 ± 0.32	95.33 ± 0.22
	**Met:** 100 ± 0.01	99.9 ± 0.01	95.78 ± 0.06	100 ± 0.03	98.9 ± 0.01	94.59 ± 0.26
**Production Yield** **(%±SD)**	99.10 ± 9.1	100 ± 6.60	99.40 ± 2.30	107.33±3.78	88±9.16	69.66±6.65
**Particle size** **(%±SD)**	-	-	-	323.59±16.98	316.22±17.78	549.54±19.50
**Absorbed moisture** **(% ± SD)**	0.79 ± 0.44	1.06 ± 0.95	4.52 ± 1.79	-	-	-
**Moisture loss** **(% ± SD)**	0.79 ± 0.04	0.73 ± 0.08	2.85 ± 0.66	-	-	-
**Surface pH** **(±SD)**	6.6 ± 0.2	6.8 ± 0.1	6.9 ± 0.1	6.8 ± 0.00	6.8 ± 0.04	6.8 ± 0.02
**Swelling index** **(%±SD)**	19.85 ± 0.02	20 ± 0.01	14.18 ± 0.02	15.67 ± 0.82	16.20 ± 0.17	17.47 ± 0.41
**Mucoadhesive strength** **(g/cm**^2^**±SD)**	134.81 ± 13.1	127.61 ± 8.4	120.50 ± 6.6	0.8 ± 0.16	1.11± 0.16	1.38 ± 0.09
**Residence time** **(min±SD)**	0.42 ± 0.002	0.33 ± 0.014	0.5 ± 0.004	61± 12.77	78 ± 19.67	130 ± 8.86

**Figure 2:**
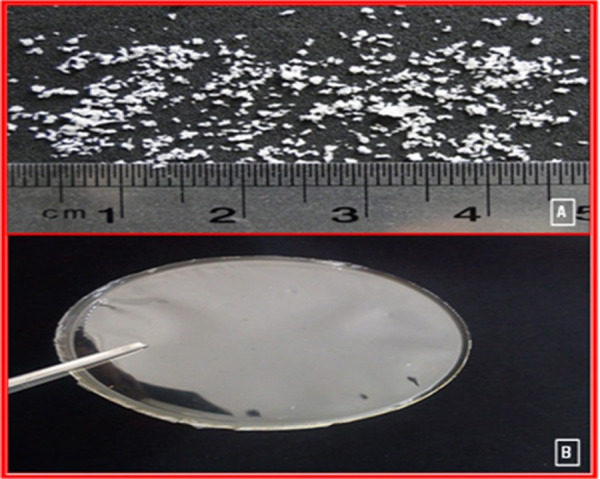
Optical microscopic photograph of mucoadhesive of sumatriptane combined with metoclopramide 2A: microparticle; 2B: film.

All the formulations were smooth, flexible, colorless (transparent), non-sticky and elegant in appearance, except for SM_3_ film (dark color) (Figure 2).

The cut films were 1x1 cm^2^ in size. The weight and thickness of SM films were in the range of 9.5-14.02 mg and 110.3-243 µm, respectively (Table 2). The SM_1_ and SM_2_ films resisted the breakage upon folding them for more than 300 times at the same place (Table 2).

The content of films was in the range of 1.03-1.80 mg/cm^2^ (Sum) and 0.59-0.57 mg/cm^2^ (Met), respectively. Though there was a small change in the loss of drug (Sum and Met) among the formulations, more uniformity was observed for films (96.63-100%,SM film, Sum combined with Met); 99.78-100%, SM' film, Met combined with Sum) as shown in Table 2.

The optical image showed that the microspheres obtained from all the formulations had almost spherical shape with smooth surface microspheres (Figure 2). Particle analysis of microspheres prepared is shown in Table 2. An increase in the ratio of polymers from 1:4:6 to 1:4:14 resulted in a significant effect on the mean particle size of microparticles. The data scrutinizing showed that all obtained microparticles adhered to a log-probability distribution. The mean particle size significantly varied according to the amounts of polymer used for the preparation of microspheres; this may be due to a difference in the viscosity of polymer solutions, as high viscosity of the polymer solution (SM_6_; 1:4:14 ratio) requires a high shearing energy to break the droplets of emulsion. Microspheres containing 700 mg HPMC were larger in comparison to other microspheres because this polymeric solution had more viscosity.

It is worth mentioning that an increase in the concentration of polymer in the internal phase leads to the increase in the size of microspheres. This occurs as at a higher concentration, polymer solution represents more viscosity which in turn requires more energy to break the droplets of dispersed phase.

Rank order of percentage drug loading for various formulations was found to be as follows: SM_4_>SM_5_>SM_6_.

Percentage drug loading efficiency of microspheres was found in the range of 54.73 to 72.24% (Table 2). Formulation SM_6_ (1:4:14 ratio) containing 50 mg Met, 200 mg Sum and 700 mg HPMC showed a maximum percentage of drug loading efficiency about 71.26% (for Sum) and 72.24% (for Met) because these microspheres had larger size as compared to other formulations. Whereas formulation SM_4_ (1:4:6 ratio) containing 300 mg HPMC showed the minimum percentage of drug loading efficiency around 54.73% (for Sum) and 60.28% (for Met), because these microspheres were small in size, which resulted in more loss of drug from surface during washing of microspheres.

The percentage of moisture absorption was shown to range from 0.79±0.44 to 4.52±1.79% for films and 15.67±0.82 to 17.47±0.41% for discs. The moisture loss measured as 1.62-1.41% (for films) is shown in Table 2. The percentage swelling of different microparticles’/discs’ formulations was shown at different time intervals. The results depicted that all microsphere formulations swelled slowly when immersed in 0.2 M phosphate buffer (pH 6.8). The percentage swelling of different microparticle formulations was found to be followed after 4 h of incubation. Percent swelling was observed to be 15.67±0.82% (SM_4_), 16.20±0.17% (SM_5_) and 17.47±0.41% (SM_6_), respectively (Table 2). The effect of drug on the swelling features of polymer is primarily associated with the substituted groups of the polymer. The hydroxyl group in the molecules plays a remarkable role in the matrix integrity of the swollen hydrophilic cellulose matrices. The matrix integrity is determined by the amount and properties of the incorporated drug. For a mucoadhesive polymer to expand and create a proper macromolecular mesh of sufficient size, the hydration is required to occur. The hydration is also longed for mobility in the polymer chains in order to enrich the interpenetration process between polymer and mucin. Polymer swelling induces a mechanical entanglement and exposes the bioadhesive sites to hydrogen bonding and/or electrostatic interaction between the polymer and the mucous network.^23^ However, the occurrence of optimum swelling and bioadhesion is accompanied by a critical degree of hydration of the mucoadhesive polymer.^13^

The variation in weight and thickness among the formulations may be observed as a result of difference in concentration of drugs (Sum and Met) used in the films (Table 2). The combination of Sum and Met Films did not show any cracks even after folding for more than 300 times. Hence it was supposed as the end point. The values were considered optimum as they revealed appropriate film properties. Numerous hydrophilic functional groups such as hydroxyl and carboxyl are present in mucoadhesive polymer (HPMC). These groups cause hydrogen to bond with the substrate (mucus), to swell in aqueous media, and thereby allow maximal exposure of potential anchor sites. Furthermore, swollen HPMC has the maximum distance between its chains. This distance results in an increased chain flexibility and efficient penetration of substrate. HPMC (with low molecular weights) either forms loose gels or dissolves quickly. Chain flexibility is a required factor in interpretation and entanglement of mucoadhesive HPMC polymer. When water-soluble polymer becomes cross-linked, the mobility of individual polymer chains reduces and in turn the effective length of the chain that is demanded for penetration into the mucous layer decreases. This reduction in turn results in the reduction of bioadhesive strength.^24,25^

The comparative percentage swelling for various formulations was in order of SM_4_> SM_5_ > SM_6_. A high percentage swelling was observed in HPMC containing microspheres due to the presence of more hydroxyl group in the HPMC molecules. The weight of these formulations was increased to the extent of 30 to 110% from the initial value within 4 h (Table 2). Although the remarkable increase in surface area during swelling could develop drug release, the increase in diffusion path length of the drug may conversely postpone the release.

As the drug was uniformly dispersed in the matrix of polymer, a significantly good amount of drug was loaded in all the formulations. Hence, the loss of drug could be related to its aqueous insolubility. Sum and Met are water-soluble and does not begin settling down from medicated solutions when dispersed for removal of air bubbles. Thus, the solutions were casted as films containing utter amount of drugs.

Percentage moisture absorption was associated with the capacity of excipients in absorbing vapor-form water. The HPMC polymer used was hydrophilic. It is believed that initial moisture content acts as distinct factor in moisture absorption. Therefore the high moisture absorbing capacity was evinced in SM_3_ (4.52%) and more moisture loss was observed in SM_1_ (1.62%). The other films primarily had high moisture content as was confirmed by percentage moisture loss. There was an inverse association between these two parameters, that is, the higher the percentage moisture loss, the lower the moisture absorption and vice versa.^12^

All formulations were of pH 6.6-6.9 for film and 6.66-6.8 for discs, respectively and it may be concluded that the films were safe and non-irritating to the oral mucosa (Table 2).

The surface pH of all discs was within the range of salivary pH (5.13-5.96). No significant difference was found in the surface pH of different films and discs (Table 2).

The acidic or alkaline pH may cause irritation to the sublingual mucosa. It may also influence the drug release and the degree of polymer hydration. Therefore it was revealed that the surface pH of sublingual film/buccal discs may optimize the drug release as well as mucoadhesion. The surface pH of all formulations was determined within ±0.5 units of the buccal pH (6.6-6.9); hence, it were anticipated no mucosal irritations and patient compliance was finally achieved.^26^

**Figure 3:**
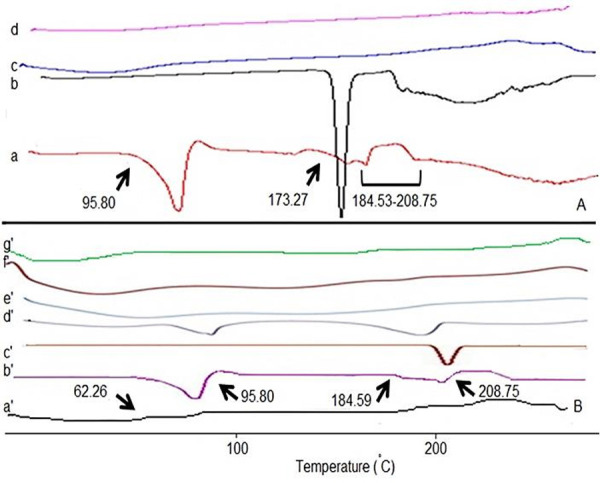
DSC thermogram of (A) film: sumatriptan (a); metoclopramide (b); HPMC (c); SM_2_ (66.7 mg sumatriptan, 25 mg metoclopramide and 200 mg HPMC) (d); (B) disc: HPMC (a'); metoclopramide (b'); sumatriptan (c'); Physical mixture SM_4_ (200 mg sumatriptan, 25 mg metoclopramide and 300 mg HPMC) (d'), SM_4_ (e'); SM_5_ (f'); SM_6_ (g').

Pure Sum exhibited a sharp melting exothermic and endothermic peak around 173.27 °C and Met was melted at 184.59-208.75°C (Figure 3). The intensity of Sum drug fusion peak, however, for the film and disc formulations was disappeared.

Pure Met monohydrate has a very high melting point (184.59-208.75°C). The endothermic peak at around 95.80 °C occurs probably due to the transition of drug to the anhydrous form via loss of one mole of water. It is clearly observed from the thermogram of the films and discs (Figure 3) that the drugs (Sum and Met) peak has been disappeared. However in the thermogram of formulations, the endothermic peak corresponding to the drugs melting was absent, suggesting the amorphous state of the drugs (Sum and Met).

The DSC analysis of films/discs revealed a significant change in the melting point of Sum and Met drugs. This change illustrated a modification or interaction between the drug and polymer (Figure 3).

*In vitro* residence time determined the period of Sum adhering combined with Met films to the mucosa and it ranged from 25-30 s. All films showed low diameter swelling and the recorded swelling index for films was 14.18-20% after 4 h.

The *in vitro* residence time with mucosa for microparticles in phosphate buffer (pH 6.8) varied from 61 to 130 min (Table 2). Microparticles showed the highest mucoadesion in this study, and did not dissolve in 0.2 M phosphate buffer (pH 6.8) for about 4h. For SM_4_ formulation, percentage of microparticles remaining was 61 min. SM_5_ and SM_6_ showed 78 and 130 min mucosa retentive time, respectively.

The results of* in vitro* bioadhesive strength study are shown in Table 2. The bioadhesion characteristics were affected by the concentration of bioadhesive polymer (HPMC). SM_1_ films containing 1:2:8 ratios (Sum:Met:HPMC ratio) indicated the highest mucoadhesivity (134.81±13.1 g/cm^2^). Conversely, SM_3_ Formulations containing 1:4:8 ratios (Sum:Met:HPMC ratio) showed the lowest mucoadhesivity (120.5±6.6 g/cm^2^).

The results of* in vitro* bioadhesive strength study for discs are also shown in Table 2. SM_6_ Formulation containing 1:4:14 ratio (Met:Sum:HPMC) showed the highest mucoadhesivity (1.38±0.09 g/cm^2^).

The integrity of Sum combined with Met films was lost early following their rapid uptake. Sum and Met drugs contain water-soluble molecules, which permit more water influx and result in quicker dissolution and erosion from mucosal surface. On the other hand, HPMC is a hydrophilic polymer and supposedly represents an affinity towards mucin comprising of 95% water. This property may be the reason for longer residence time (integrity of the films is lower). As it was also reported by several previous studies, the enhanced erosion rate was observed in some non-ionic polymers such as HPMC. The swelling behavior was assessed by measuring the diameter swelling. In the case of SM films supplied for sublingual (local) therapy uses, the contact area should have been as large as possible. This demand had to be balanced with patient compliance. Moreover, any excessive increase in the film diameter might have caused discomfort and/or dislodgment of the swollen film (lower than 20% swelling for Sum combined with Met films). In an *in vitro* mucoadhesion test performed by Nakanishi *et al.*,^27^ the mucoadhesion force was dependant on the hydrogen bond made between the hydroxyl group in the polymer and mucus. It formed an ionic complex with hyaluronic acid that provided higher binding power. Bio-adhesive strength of formulation was increased due to a rise in the ratio of polymer. In the mucoadhesion process, this increase is required for swelling and expansion of the polymer chain as the interpenetration and entanglement of the polymer and the mucous networks supposedly are responsible for the adhesion.^28,29^ Therefore, it seems that bioadhesives should swell and expand swiftly having contacted with water. For the hydrophilic polymer, hydration is responsible for the adhesion of polymer to the mucous membrane; as hydration makes these polymers sticky and hence they adhere to the mucous membrane. A high percentage of adhesion indicates that microspheres have excellent mucoadhesion to the mucosal tissue. HPMC interacts with the mucin and results in the adhesion of polymer to the mucin. The formulation films showed the highest mucoadhesivity, while the formulation discs indicated the lowest mucoadhesion strength due to the small size of microsphere which takes short time for solubilization. In this study, it was found no correlation between the bioadhesion force and the residence time of HPMC polymer. It seems that the high bioadhesive polymers do not necessarily inhabit any longer on the mucosal surface. Surface charge density and chain flexibility are supposed to be preconditions for bioadhesion, whereas the residence time is basically dealt with the dissolution rate of the polymer.^30-32^

**Figure 4:**
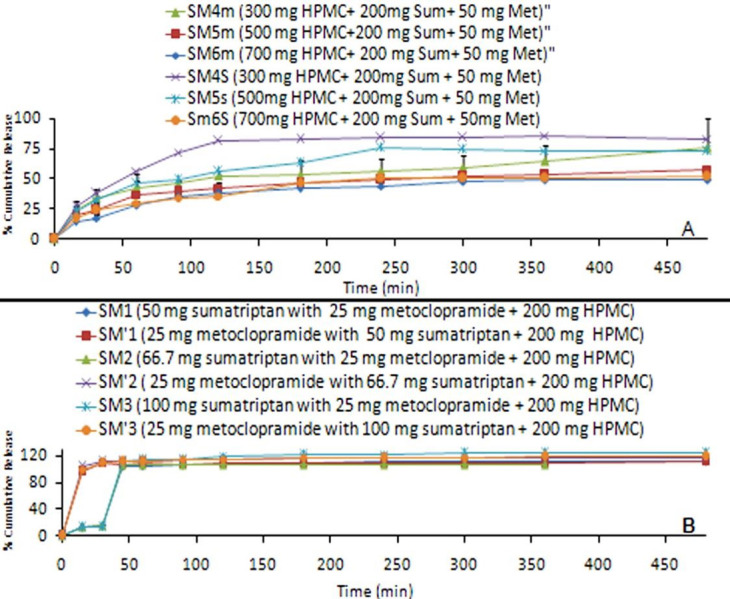
Cumulative percentage release of sumatriptan combined with metoclopramide from (A) films and (B) discs prepared with different drug to polymer ratios.

The release profiles for all films are showed in Figure 4. Films with high content uniformity or high drug entrapment showed a faster dissolution rate. Since more drugs are released from the films, it is supposed that more channels and pores are produced, contributing to the faster drug release rates. Comparison of various dissolution profiles is analyzed by several special measures including the dissolution Rel_0.5_ (amount of drug release after 0.5h), Rel_0.75_ (amount of drug release after 0.75h), Rel_8_ (amount of drug release after 8h), efficiency (DE %) and the difference factor (F_1_). The difference factor is used to determine whether the test product is different to the reference products (SM_1_ formulation (1:2:8 ratio) is selected as reference formulation).

Accordingly, Figure 4 shows that the initial Sum drug releases (Rel_0.25_) for the SM_1_ to SM_3_ formulations were low (12.60%, 12.42% and 12.67%, respectively) and Rel_0.5_ was 103.10%, 105.82% and 112.57%, respectively. Moreover, Met drug release of SM’ (SM) films shows that high burst effect for SM’_1_ to SM’_3_ formulations were high (96.10%, 102.83% and 99.43%, respectively) and Rel_0.5_ was high, too (106.68%, 110.82% and 110.8%, respectively) (Figure 4B). The release of Met drug from SM films was faster than the release of Sum drug from SM films (*p*<0.05). During dissolution, HPMC containing films swelled forming a gel layer on the exposed mucous surfaces.

The release of drug from Sum combined with Met films with an increase in Sum concentration, and the interaction between the polymer and drug increased with the formation of a closer network, showed an increase in the diffusion of drug from the films (Figure 4A & 4B). The reason for the burst release (Rel_0.25_) could be due to the presence of some pores and channels of polymer near to the surface of films. When water-soluble drugs (Sum and Met) did not show a tendency to migration or removal of air bubbles, thereby drug concentration is increased in the films and burst effect is induced.^12^ The pores present in HPMC polymer act as channels for the entrance of liquid medium through the film surface and cause it to swell. Hydrogen bond between the hydroxyl groups of HPMC moiety and mucous surface decreases its porosity and permeability. Thus, if the ratio of drug to polymer be varied, the rate of release of drug could be controlled. Dissolution medium permeation into the films is facilitated due to the high swelling action of polymer which leads to more medium for the transport of the drug available. While the discs showed the least drug release, the drug release from microsphere was significantly affected by the size of microspheres. The increase in the polymer concentration led to an increase in the size of microspheres thus drug release from microspheres. Hence drug to polymer ratio was found to significantly decrease. SM_6_ Formulation (1:4:14 ratio) showed the fastest drug release among all the disc formulations as these microspheres are small in size.

logP values for Sum and Met were 0.67 and 2.76, exhibiting low permeability through buccal mucosa. Akin to other studies, the obtained results indicated that generally an increase in the ratio of drug to polymer resulted in a reduction in the release of Sum combined with Met from discs (Table 3 and Figure 4).^33^

**Table 3:** Flux or amount of drug release per unit surface area after 4 h, permeability coefficient and regression coefficient for different formulation and comparison of various release characteristics of sumatriptan combined with methoclopramide from different film/disc formulations

**Formulation code**		^a^**Rel**_0. 5_ **(%±SD)**	^b^**Rel**_0.75_ **(%±SD)**	^c^**Rel**_8_ **(%±SD)**	^e^**DE** **(%±SD)**	^f^**t**_50%_ **(min±SD)**	^g^**f**_1_	**Flux** **[(mg/cm**^2^**.min)/10**^3^**]**	**Permeability coefficient** **[(cm/min)/10**^3^**±SD]**	**r**^2^
**SM**_1_	**S**_1_	15.86±3.27	103.41±2.90	111.59±15.30	100.26±12.32	49.08±5.47	0	1.82±0.003	18.2±1.11	0.872
	**M**_1_	109.87±3.71	106.67±3.04	110.75±4.71	93.85±10.24	138.16±14.52	0	1.46±0.002	50.34±7.45	0.940
**SM**_2_	**S**_2_	15.41±2.72	105.82±3.73	106.45±4.08	104.61±9.87	49.35±4.12	4.94	1.62±0.002	12.9±0.82	0.969
	**M**_2_	116.63±1.72	110.82±1.54	117.80±5.06	106.37±12.14	143.71±14.78	14.80	1.23±0.001	38.44±5.63	0.959
**SM**_3_	**S**_3_	13.47±1.34	112.57±4.78	123.86±5.44	100.32±13.12	54.22±4.78	2.46	1.47±0.001	8.65±0.89	0.931
	**M**_3_	108.54±7.03	110.80±1.59	118.23±4.42	116.60±14.39	71.23±6.25	23.94	1.02±0.0005	18.55±1.23	0.884
**SM**_4_	**S**_1_	37.99±3.30	56.74±6.32	83.28±1.19	92.77±13.32	54.01±4.96	13.30	1±0.0004	35.71±4.65	0.935
	**M**_1_	37.30±7.11	42.24±12.33	75.49±25.38	66.94±5.64	106.28±6.25	32.28	0.8±0.0003	33.33±6.52	0.994
**SM**_5_	**S**_2_	32.7±4.06	46.02±4.48	72.62±0.80	55.32±5.98	52.82±3.69	46.48	0.8±0.0002	18.60±1.27	0.983
	**M**_2_	23.74±6.57	37.17±11.87	58.21±1.65	56.43±4.25	176.75±25.14	43.66	0.72±0.0003	14.40±1.86	0.975
**SM**_6_	**S**_3_	24.01±3.77	29.91±2.02	51.62±0.56	48.36±6.21	54.29±3.69	52.74	0.65±0.0003	9.29±0.56	0.973
	**M**_3_	17.30±3.46	27.83±3.92	49.44±0.27	40.72±2.17	113.63±21.10	61.38	0.45±0.0002	5.63±0.32	0.920

^a^ Rel_0.25_ = amount of drug release after 15 min; ^b^ Rel_.75_ = amount of drug release after 30 min; ^c^ Rel_8_ = amount of drug release after 8h ;^d^DE = dissolution efficiency; ^e^t 50% = dissolution time for 50% fractions; ^f^ f_1_ = Differential factor (0<f_1_<15), SM_1_(1:2:8 ratio) is selected as reference formulation.

Figure 4A draws a comparison of permeation of Sum combined with Met films and discs through sheep buccal mucosa for formulations containing different drug to polymer ratios. Slopes of the linear portion of release profiles were calculated. These slopes portrayed the rate of release or flux of Sum combined with Met from different formulations (Table 3). The highest fluxes and regression coefficient for films as SM_1_ (reference formulation) formulations were 0.00182 mg/cm^2^.min, 0.972 (Sum), and 0.00146 mg/cm^2^.min, 0.940 (Met), respectively.

Furthermore, Figure 4B draws a comparison of permeation of Sum and Met through buccal mucosa for formulations containing different drug to polymer ratios. Slopes of the linear portion of the release profiles were calculated. These slopes depicted the rate of release or flux of Sum and Met from different formulations (Table 3). The highest fluxes and regression coefficient for discs as SM_4_ formulations were 0.001 mg/cm^2^.min, 0.995 (Sum), and 0.0008 mg/cm^2^.min, 0.994 (Met), respectively.

The pores present in HPMC polymer act as channels for the entrance of liquid medium through the microparticles wall and cause it to swell. The hydrogen bond between the hydroxyl groups of the carboxylic moiety and the carbonyl oxygen of ester group increases the degree of solidity of polymer and decreases its porosity and permeability. Thus, by varying the ratio of drug to polymer the release rate of Sum and Met could be controlled.

**Figure 5:**
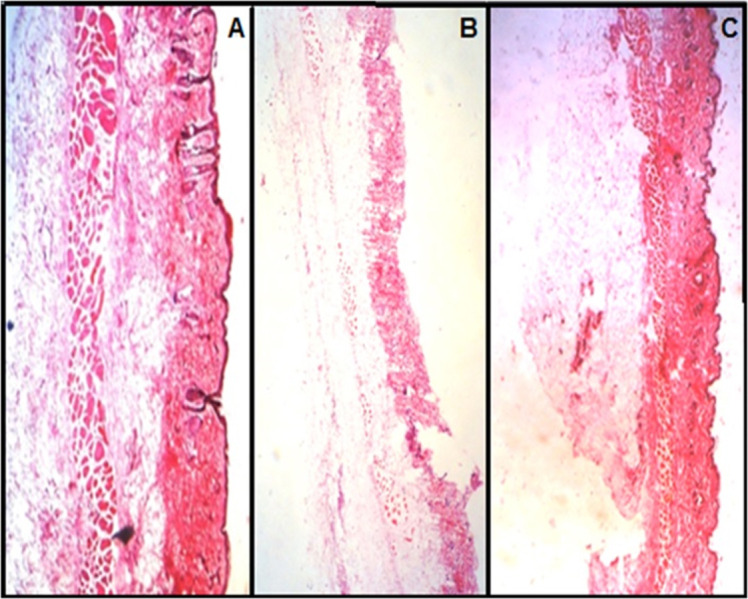
Histopathological evaluation of sections of buccal mucosal areas (A) un-treated (B) treated with film (C) treated with disc containing sumatriptan combined with methoclopramide (magnitude X).

The microscopic observations indicated that none of the films and discs had a significant effect on the microscopic structure of mucosa. As shown in Figure 5, no cell necrosis was observed.

Cellular membrane was intact and no damage to the treated buccal mucosa was observed (was used in the bioadhesion strength test). Thus, the formulation containing microparticles appeared to be safe with respect to the buccal administration (Figure 5A, 5B & 5C).

## Conclusion

In recent years, interest has been grown to develop a drug delivery system with the use of a mucoadhesive polymer (e.g., HPMC) that could attach to the related tissue or to the surface coating of the tissue to target various absorptive mucosa, buccally or sublingually. The mucoadhesive polymers could themselves exert some control over the rate and amount of drug release and thus contribute to the therapeutic efficacy of mucoadhesive drug delivery system. The buccal drug delivery system bypasses the liver and avoids the pre-systemic elimination in the gastrointestinal tract and increases bioavailability. The mucosa is relatively permeated with a rich blood supply. Two buccal and sublingual formulations of HPMC were prepared with HPMC polymer. Therefore, in the present study an attempt was made to study the mucoadhesive strength of different dosage forms by a straightforward *in vitro* method. Among the various Sum and Met combinations, the SM_3_ formulation (100 mg Sum, 25 Met and 200 mg HPMC) was found to be the most suitable. The formulation SM_4_ fulfilled the requirement of a proper buccal disc. The force of adhesion (g/cm^2^) of different forms was found in the order of film > disc. The films showed the highest swelling as well as the highest mucoadhesive strength. It showed *in vitro* residence time up to 2.2 h. It followed *in vitro* drug release up to 112.57 % for 45 min and *in vitro* drug permeation up to 4 h. *In vitro* release studies of Sum and Met microparticles showed a significant difference in their release rate. The microparticles prepared showed a slow and controlled release over lengthened period of time when compared to the other mucoadhesive films. These results may suggest the potent application of films and microparticles as a suitable sustained release drug delivery system. These mucoadhesive films could be exploited in the effective and promising development of mucoadhesive drug delivery systems.

## Acknowledgments

The financial support from Drug Applied Research Center and Research Council of Tabriz University of Medical Sciences is greatly acknowledged.

## Ethical Issues

Not applicable.

## Conflict of Interest

Authors certify that no actual or potential conflict of interests exists in relation to this article.
